# Regenerative effects of human embryonic stem cell‐derived neural crest cells for treatment of peripheral nerve injury

**DOI:** 10.1002/term.2642

**Published:** 2018-02-18

**Authors:** Iwan Jones, Liudmila N. Novikova, Lev N. Novikov, Monika Renardy, Andreas Ullrich, Mikael Wiberg, Leif Carlsson, Paul J. Kingham

**Affiliations:** ^1^ Umeå Center for Molecular Medicine Umeå University Umeå Sweden; ^2^ Laboratory of Neural Repair and Cellular Therapy, Department of Integrative Medical Biology Umeå University Umeå Sweden; ^3^ ITV Denkendorf Product Service GmbH Denkendorf Germany; ^4^ Hand and Plastic Surgery, Department of Surgical and Perioperative Sciences Umeå University Umeå Sweden

**Keywords:** artificial nerve graft, human embryonic stem cells, neural crest cells, peripheral nerve injuries, peripheral nervous system

## Abstract

Surgical intervention is the current gold standard treatment following peripheral nerve injury. However, this approach has limitations, and full recovery of both motor and sensory modalities often remains incomplete. The development of artificial nerve grafts that either complement or replace current surgical procedures is therefore of paramount importance. An essential component of artificial grafts is biodegradable conduits and transplanted cells that provide trophic support during the regenerative process. Neural crest cells are promising support cell candidates because they are the parent population to many peripheral nervous system lineages. In this study, neural crest cells were differentiated from human embryonic stem cells. The differentiated cells exhibited typical stellate morphology and protein expression signatures that were comparable with native neural crest. Conditioned media harvested from the differentiated cells contained a range of biologically active trophic factors and was able to stimulate *in vitro* neurite outgrowth. Differentiated neural crest cells were seeded into a biodegradable nerve conduit, and their regeneration potential was assessed in a rat sciatic nerve injury model. A robust regeneration front was observed across the entire width of the conduit seeded with the differentiated neural crest cells. Moreover, the up‐regulation of several regeneration‐related genes was observed within the dorsal root ganglion and spinal cord segments harvested from transplanted animals. Our results demonstrate that the differentiated neural crest cells are biologically active and provide trophic support to stimulate peripheral nerve regeneration. Differentiated neural crest cells are therefore promising supporting cell candidates to aid in peripheral nerve repair.

## 1 INTRODUCTION

The peripheral nervous system (PNS) has an innate repair capacity, but this process is seldom successful, and surgical intervention following injury is almost always required. The current gold standard treatments are either direct microsurgical nerve repair or autologous nerve grafts. However, the restoration of motor and sensory modalities often remains incomplete because full functional recovery is dependent upon many factors including the age of the patient, injury severity, and trauma location. The development of alternative repair strategies that either complement or even replace current established surgical procedures is therefore warranted (Wiberg & Terenghi, 2003).

The current ability to manufacture artificial nerve grafts makes them an alternative therapeutic approach for peripheral nerve repair. Modern grafts are composed of biodegradable conduits that act as guidance scaffolds into which biochemical cues are incorporated, which may include supporting cells, growth factors, and extracellular matrix components (Gu, Ding, & Williams, 2014). Previous studies have demonstrated that artificial nerve grafts seeded with supporting Schwann cells can enhance axonal regeneration in animal models (Mosahebi, Fuller, Wiberg, & Terenghi, 2002). However, the routine clinical use of Schwann cells is somewhat restricted because their isolation requires invasive biopsies, and they display limited *in vitro* expansion capability (Gu et al., 2014). Therefore, one of the current goals of regenerative medicine is to identify Schwann cell‐like candidates that could act as supporting cells in an artificial nerve graft.

Embryonic stem cells (ESC) are one possible candidate because they are infinitely renewable and amenable to molecular manipulation (Fairbairn, Meppelink, Ng‐Glazier, Randolph, & Winograd, 2015). A previous study demonstrated the usefulness of mouse ESC‐derived neuronal progenitors for the treatment of peripheral nerve injuries (PNI; Cui et al., 2008). Despite these encouraging results, very little is known about the efficacy of human ESC (hESC)‐derived supporting cells in artificial nerve graft models for the treatment of PNI. This is surprising because a number of studies have demonstrated that hESC can be differentiated into neural crest cells (NCCs) and associated PNS lineages (Lee et al., 2007; Pomp, Brokhman, Ben‐Dor, Reubinoff, & Goldstein, 2005; Ziegler, Grigoryan, Yang, Thakor, & Goldstein, 2011).

In this study, we assess the efficacy of hESC‐derived NCCs in artificial nerve grafts. We demonstrate that the differentiated NCCs are able to provide trophic support and stimulate both *in vitro* neurite outgrowth and *in vivo* sciatic nerve regeneration. The promising results achieved in this study demonstrate that differentiated NCCs are potential candidates as renewable supporting cells and should be considered as an alternative source to Schwann cells in artificial nerve graft approaches for the treatment of PNI.

## 2 MATERIALS AND METHODS

### 2.1 Ethical statement

All experiments involving animals were approved by the Animal Review Board at the Court of Appeal of Northern Norrland in Umeå (DNR #A186‐12).

### 2.2 Cell culture

hESCs (H9, WA09, WiCell Research Institute) were cultured on feeder layers of irradiated CF‐1 mouse embryonic fibroblasts (Jackson Laboratory) in Dulbecco's modified Eagle's medium (DMEM)/F12 (Thermo Fisher Scientific) supplemented with 20% (vol/vol) KnockOut Serum Replacement (Thermo Fisher Scientific), 1× Non‐Essential Amino Acids (Thermo Fisher Scientific), 100 mM L‐glutamine (Sigma‐Aldrich), 0.1 mM β‐mercaptoethanol (Sigma‐Aldrich), 1% (vol/vol) Penicillin–Streptomycin (PeSt; Thermo Fisher Scientific), and 4 ng/ml basic fibroblast growth factor (bFGF; Thermo Fisher Scientific). Cultures were enzymatically passaged onto new CF‐1 mouse embryonic fibroblasts using collagenase IV (Thermo Fisher Scientific).

SH‐SY5Y neuroblastoma cells (Advanced Tissue Culture Collection) were cultured in standard tissue culture vessels in DMEM (Thermo Fisher Scientific) supplemented with 10% (vol/vol) heat‐inactivated fetal calf serum (FCS; Sigma‐Aldrich) and 1% (vol/vol) PeSt. Cultures were enzymatically passaged using trypsin (Thermo Fisher Scientific).

Primary sensory neurons were dissociated from the dorsal root ganglia (DRG) of adult female Sprague Dawley rats (Taconic Biosciences; Tse, Novikov, Wiberg, & Kingham, 2015). The isolated neurons were seeded onto poly‐D‐lysine (Sigma‐Aldrich) and laminin (Sigma‐Aldrich)‐coated culture vessels in DMEM/F12 supplemented with 1 mg/ml bovine serum albumin (Sigma‐Aldrich), 10 μM cytosine arabinoside (Sigma‐Aldrich), 10 pM insulin (Sigma‐Aldrich), 100 μM putrescine (Sigma‐Aldrich), 30 nM sodium selenite (Sigma‐Aldrich), 20 nM progesterone (Sigma‐Aldrich), and 0.1 mg/ml apo‐transferrin (Sigma‐Aldrich).

Schwann cells were isolated from the sciatic nerves of adult female Sprague Dawley rats (Reid et al., 2011). Cells were grown on poly‐D‐lysine‐coated culture vessels (Thermo Fisher Scientific) in DMEM supplemented with 10% (vol/vol) heat‐inactivated FCS, 50 ng/ml neuregulin (R&D Systems), and 10 μM forskolin (Sigma‐Aldrich). Cultures were enzymatically passaged using trypsin.

### 2.3 Differentiation of NCCs from hESCs

NCCs were differentiated from hESCs by a combination of dual‐SMAD inhibition and early WNT activation (Chambers, Mica, Lee, Studer, & Tomishima, 2016). After completion of the differentiation phase, the NCC population was enriched by magnetic‐activated cell sorting (MACS) using a CD271 human MicroBead Kit (nerve growth factor receptor [NGFR]; Miltenyi Biotec). The sorted NCCs were then plated on matrigel‐coated culture vessels (Corning) in N2 media (Thermo Fisher Scientific) supplemented with 10 ng/ml bFGF and 20 ng/ml epidermal growth factor (EGF; R&D Systems). Cultures were enzymatically passaged onto new matrigel‐coated plates by accutase (Innovative Cell Technologies) treatment for a maximum of five passages over a period of 10 days.

### 2.4 Immunocytochemistry

NCCs were plated onto matrigel‐coated coverslips and cultured in N2 medium supplemented with 10 ng/ml bFGF and 20 ng/ml EGF. The cells were incubated for 48 hr for characterisation studies and 5 days for spontaneous differentiation analysis. Immunocytochemistry was performed as previously described (Kingham, Kolar, Novikova, Novikov, & Wiberg, 2014). The following primary antibodies were used: transcription factor AP‐2 alpha (TFAP2A; DHSB, mouse monoclonal, 1:100), glial fibrillary acidic protein (Dako, rabbit polyclonal, 1:400), β‐1,3‐glucuronyltransferase 1 (B3GAT1; Sigma‐Aldrich, mouse monoclonal, 1:100), microtubule‐associated protein 2 (Chemicon, rabbit polyclonal, 1:100), TUBB3 (Sigma‐Aldrich, rabbit polyclonal, 1:500), NEFH (Abcam, mouse monoclonal, 1:500), NGFR (Advanced Targeting Systems, mouse monoclonal, 1:100), S100 calcium‐binding protein B (S100B; Dako, rabbit polyclonal, 1:2000), and SRY‐box 10 (SOX10; R&D Systems, mouse monoclonal, 1:100). The specificity of NGFR antibody was validated by immunocytochemical staining of both hESCs‐ and MACS‐enriched cells. NGFR positive cells were only observed in the enriched population, thus confirming the specificity of the NGFR antibody for NCCs as has been previously documented (Lee et al., 2007). No NGFR positive cells were observed in the hESCs cultures.

### 2.5 Reverse transcription–polymerase chain reaction

Total RNA was isolated using the RNeasy™ kit (Qiagen) and cDNA was synthesised using the SuperScript™ First Strand Synthesis System (Thermo Fisher Scientific). Polymerase chain reaction (PCR) reactions were amplified from 10 ng cDNA templates using the GoTaq® Green Master Mix (Promega). PCR amplicons were resolved on 2% (wt/vol) agarose gels and documented using a Gel Doc 2000 (BioRad). Quantitative reverse transciption–PCR was performed as previously described (Kingham et al., 2014). Please see the Supporting Information section for all primer sequences and amplicon sizes.

### 2.6 Preparation of conditioned medium

NCCs were plated onto matrigel‐coated flasks and cultured in N2 medium supplemented with 10 ng/ml bFGF and 20 ng/ml EGF. hESCs were seeded as monolayers (Chambers et al., 2016) on matrigel‐coated culture vessels in DMEM/F12 supplemented with 20% (vol/vol) KnockOut Serum Replacement, 1× Non‐Essential Amino Acids, 100 mM L‐glutamine, 0.1 mM β‐mercaptoethanol, 1% (vol/vol) PeSt, and 4 ng/ml bFGF. Conditioned medium was harvested after 48 hr and filtered using a Steriflip^®^ Filtration System (Millipore).

### 2.7 Enzyme‐linked immunosorbent assay

Enzyme‐linked immunosorbent assays were performed on hESC‐ and NCC‐conditioned medium using RayBio^®^ Sandwich enzyme‐linked immunosorbent assay kits (RayBiotech, Inc.).

### 2.8 Neurite outgrowth assay

SH‐SY5Y neuroblastoma cells were plated onto gelatin‐coated coverslips (Sigma‐Aldrich) and cultured for 72 hr in DMEM supplemented with 10% (vol/vol) heat‐inactivated FCS, 10 μΜ all‐trans retinoic acid (Sigma‐Aldrich), and 1% (vol/vol) PeSt. The cultures were subsequently washed in phosphate‐buffered saline (Thermo Fisher Scientific), and conditioned media was then added, and the cultures were incubated for 48 hr. Primary sensory DRG neurons seeded on poly‐D‐lysine‐ and laminin‐coated coverslips were also incubated in conditioned media for 48 hr. Cells were then processed for immunocytochemistry and neurite outgrowth analysis (Kingham et al., 2014). A minimum of 80 SH‐SY5Y neuroblastoma cells and 20 primary sensory neurons were measured for all conditions during each replicate experiment (*n* = 3).

### 2.9 Sciatic nerve injury model

Tubular conduits were manufactured from trimethylene carbonate ε‐caprolactone block‐copolymer (Lietz et al., 2006). The lumen of the conduits were filled with dilute solutions of fibrin matrix (Tisseel, Baxter; Kalbermatten et al., 2008) containing either (a) rat Schwann cells at passage 4 used as a regenerative efficacy control group, (b) differentiated NCCs (at passage 3), or (c) hESCs (at passage 37). All conduits were seeded with one million of the respective cells. Surgery was performed as previously described on adult female Sprague Dawley rats (Taconic Biosciences; 16 weeks old, *n* = 6 per treatment group) under isoflurane anaesthesia (Kingham et al., 2014). Postoperative animals were housed alone for 2 weeks, and immunosuppression of all the groups was administered daily by subcutaneous injections of cyclosporine‐A (1.5 mg/100 g body weight; Novartis). This has been demonstrated to improve sciatic nerve regeneration efficiency (Amniattalab & Mohammadi, 2017; McGrath et al., 2012; Mohammadi, Heydarian, & Amini, 2014), and we therefore included cyclosporine‐A treatment for all groups. The animals were killed 2 weeks after surgery with an intraperitoneal overdose of sodium pentobarbital (240 mg/kg body weight; Apoteketsbolaget). Conduits were harvested and fixed with 4% (wt/vol) paraformaldehyde (Sigma‐Aldrich) in 0.1 M phosphate buffer (pH 7.4) overnight at 4 °C. The conduits were then sequentially cryoprotected in 10%, 20%, and 30% (wt/vol) sucrose in 0.1 M phosphate buffer (pH 7.4) for 3 days and then embedded in OCT compound (HistoLab) at −80 °C before cryosectioning. Ipsilateral DRG and lumbar (L) 4–L6 spinal cord segments were removed, and the ventral halves of the spinal segments were dissected and snap frozen in liquid nitrogen.

### 2.10 Histology and immunohistochemistry

Histological and immunohistochemical staining was performed on conduit cryosections (16 μm) as previously described (Jones et al., 2015). The following primary antibodies were used: human nuclear antigen (HNA; Millipore, mouse monoclonal, 1:100), TUBB3 (Sigma‐Aldrich, rabbit polyclonal, 1:500), and S100B (Dako, rabbit polyclonal, 1:2000). The specificity of the HNA antibody was validated by immunohistochemical staining of conduit cryosections harvested from all PNI treatment groups. HNA positive cells were only observed in the hESC and NCC transplanted animals, thus confirming the specificity of the HNA antibody to cells of human origin. No HNA positive cells were observed in the rat Schwann cell treatment group (Figure S1).

### 2.11 Image analyses

Images were captured using a Zeiss LSM 710 confocal microscope or Nikon Eclipse E800 microscope fitted with a Nikon DS‐Ri1 digital colour camera. Images were analysed and compiled using Fiji (Schindelin et al., 2012), Adobe Photoshop, and Adobe Illustrator. The molecular marker quantification data were calculated from a minimum of 10 randomly chosen objective fields sampled from replicate differentiation experiments (*n* = 3). The percentage of molecular marker positive to DAPI positive cells was subsequently quantified. Axon profile numbers were counted using an optical microgrid. Every fifth section was first scanned to identify the sections that contained the longest axons, and three sections within the selected range were subsequently used for analysis. The number of axonal profiles was counted in a line perpendicular to the direction of the conduit in serial high power fields beginning 1 mm distally from the proximal stump and then subsequently every 500 μm until the field contained no more positive TUBB3 staining.

### 2.12 Statistical analyses

Statistical analyses were performed using Prism7 (GraphPad Software). Mann–Whitney *U*‐tests and one‐way analysis of variance followed by Bonferroni's multiple comparison tests were used to determine all statistical significances. Error bars in all figures represent the standard deviation. The *p* values are indicated as follows: ns = not significant, ^*^ *p* ≤ .05, ^**^ *p* ≤ .01, ^***^ *p* ≤ .001, and ^****^ *p* ≤ .0001.

## 3 RESULTS

### 3.1 Generation of NCCs from hESCs using a combination of dual‐SMAD inhibition and early WNT activation

**Figure 1:**
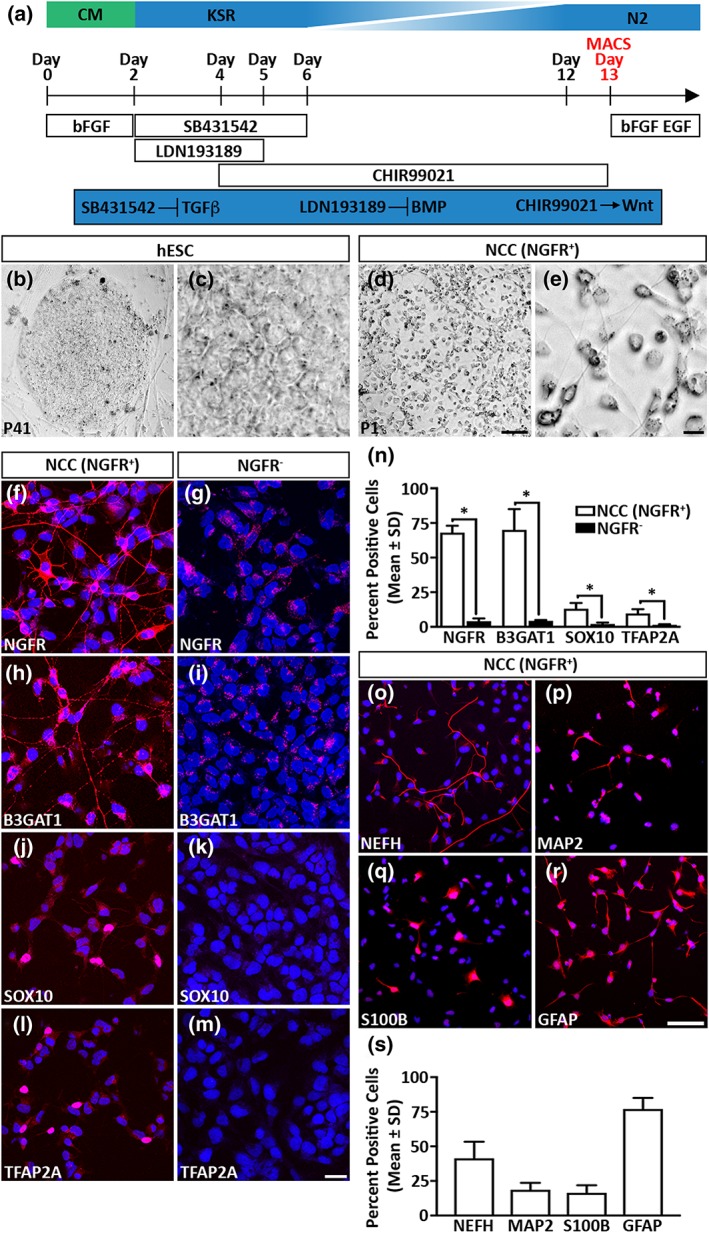
Derivation of neural crest cells (NCCs) from human embryonic stem cells (hESCs). (a) Schematic diagram outlining the differentiation protocol employed in this study. (b–e) hESC and NCC culture morphology. (f–n) Differentiated NCCs (NGFR^+^) are enriched in neural crest markers compared with the NGFR negative (NGFR^−^) cell population. (o–s) Differentiated NCCs are multipotent and can differentiate into cells that express established neuronal and glial cell markers. All data represent the mean ± *SD* of three independent experiments. Scale bars: (b, d) 100 μm, (c, e) 0 μm, (f–m) 25 μm, and (o–r) 100 μm. The *p* values are denoted as follows: ^*^ *p* ≤ .05. CM = conditioned medium; KSR = KnockOut serum replacement

The broad fate potential of NCCs makes them potential supporting cell candidates in artificial nerve graft approaches for the treatment of PNI. However, the transient nature of NCCs makes their isolation from animal models for such studies a difficult task. We therefore differentiated NCCs from hESCs by a combination of dual‐SMAD inhibition and early WNT activation (Figure 1a). Following differentiation, we isolated the neural crest population by MACS using an antibody directed against the NGFR (Lee et al., 2007). The NGFR positive population displayed stellate morphology that was very different from the original appearance of the hESC cultures (Figure 1b–e) and was highly enriched for NGFR (68% ± 5%) compared with the NGFR negative population (4% ± 2%; Figure 1f–g and n). We also observed that the NGFR positive population was also enriched for other multipotent neural crest markers: B3GAT1 (70% ± 15%), SOX10 (13% ± 5%), and TFAP2A (10% ± 3%) compared with the NGFR negative population (B3GAT1 4% ± 1%, SOX10 2% ± 1%, and TFAP2A 2% ± 1%, respectively; Figure 1h–n; Kim, Lo, Dormand, & Anderson, 2003; Lee et al., 2007; Nagase et al., 2003).

A signature feature of native NCCs is their capability to differentiate into both neuronal and glial PNS lineages (Bronner‐Fraser, 1994). Therefore, the multipotent capability of the NGFR positive cell population was verified by using a spontaneous differentiation approach (Kreitzer et al., 2013). We observed that subsets of the NGFR positive population could differentiate into cells that expressed the neuronal markers neurofilament heavy (NEFH, 41% ± 12%) and microtubule‐associated protein 2 (19% ± 5%; Figure 1o–p and s; Blanchard et al., 2015). We also observed that the NGFR positive population differentiated into cells that were expressing the glial cell markers S100B (16% ± 6%) and glial fibrillary acidic protein (77% ± 8%; Figure 1q–s; Liu et al., 2012). Taken together, our combined analyses established that differentiating hESCs by a combination of dual‐SMAD inhibition and early WNT activation followed by NGFR enrichment resulted in the isolation of *in vitro*‐derived NCCs. Furthermore, the differentiated NCCs could generate both neuronal and glial lineages.

### 3.2 Differentiated NCCs stimulate *in vitro* neurite outgrowth

**Figure 2:**
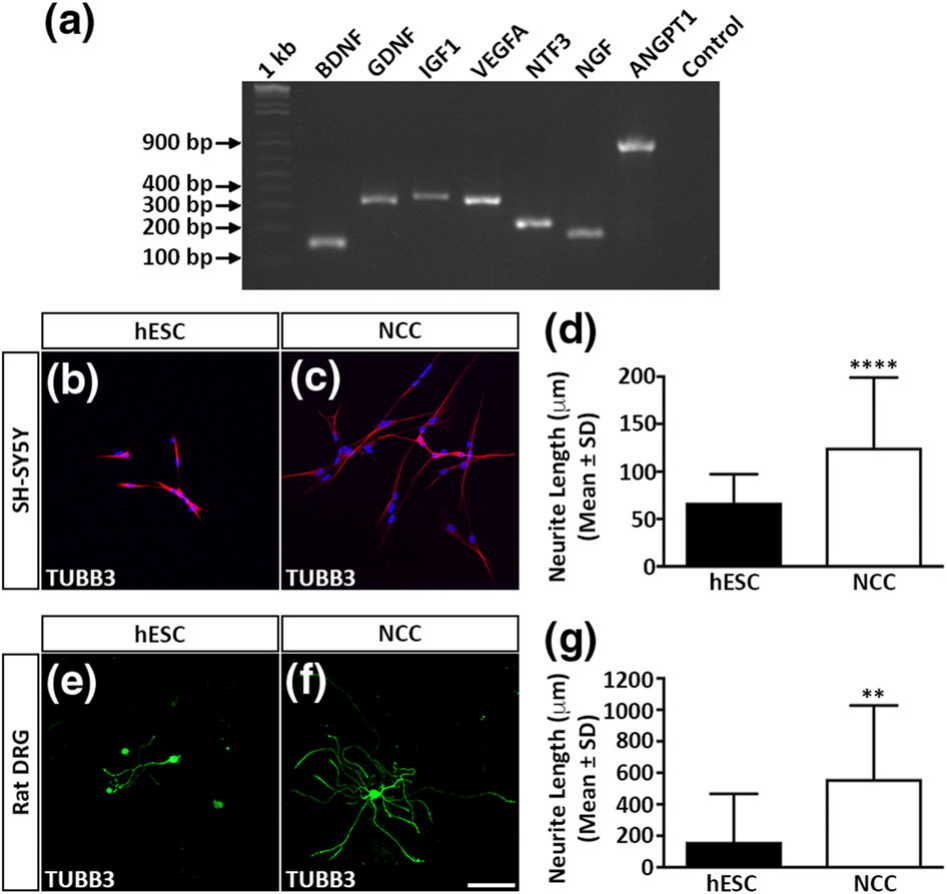
Differentiated neural crest cells (NCCs) enhance neurite outgrowth. (a) reverse transcription–polymerase chain reaction analysis of trophic factor gene expression in differentiated NCCs. (b–c) SH‐SY5Y neurite outgrowth following incubation in human embryonic stem cells (hESC)‐ or NCC‐conditioned medium. (d) Quantitative analysis of SH‐SY5Y mean neurite outgrowth. (e–f) Rat dorsal root ganglion (DRG) neurite outgrowth following incubation in hESC‐ or NCC‐conditioned medium. (g) Quantitative analysis of rat DRG mean neurite outgrowth. All data represent the mean ± *SD* of three independent experiments. Scale bars: (b–c and e–f) 100 μm. The *p* values are denoted as follows: ^**^ *p* ≤ .01, ^****^ *p* ≤ .0001

One of the main criteria of an artificial nerve graft is that the transplanted cells must provide trophic support that accelerates PNS regeneration. We therefore assessed the suitability of the differentiated NCCs for this task by analysing their trophic factor expression profile (Figure 2). We observed that the cells expressed transcripts that encoded for (a) neurotrophic factors including brain‐derived neurotrophic factor (BDNF), glial cell line‐derived neurotrophic factor, neurotrophin‐3, nerve growth factor, and insulin‐like growth factor‐1 and (b) angiogenic factors such as vascular endothelial growth factor‐A (VEGF‐A) and angiopoietin‐1 (Figure 2a). To determine if the observed gene expression patterns correlated with enhanced trophic factor secretion, we measured the protein levels in conditioned medium harvested from the differentiated NCCs. The conditioned medium contained detectable levels of BDNF (8.5 ± 0.2 ng/ml) and VEGF‐A (214 ± 4.5 pg/ml) proteins whose levels were substantially greater than those observed in conditioned medium harvested from hESCs (BDNF 0.71 ± 0.04 ng/ml and VEGF‐A 142.7 ± 1.9 pg/ml, respectively).

*In vitro* neurite outgrowth assays were subsequently used to confirm bioactivity of the secreted trophic factors. Exposure to NCC‐conditioned medium induced human SH‐SY5Y neuroblastoma cells to extend neurites (124.9 ± 74.1 μm) that were significantly longer than neuroblastoma cells treated with hESC‐conditioned medium (67.2 ± 30.3 μm; Figure 2b–d). In a similar manner, rat primary DRG neurons grown in NCC‐conditioned medium elaborated neurites (561.1 ± 466.2 μm) that were also significantly longer than the corresponding outgrowth of cells cultured in hESC‐conditioned medium (161.1 ± 305.8 μm; Figure 2e–g). In conclusion, the differentiated NCCs produced and secreted several trophic factors that were biologically active and could enhance *in vitro* neurite outgrowth.

### 3.3 Differentiated NCCs stimulate *in vivo* sciatic nerve regeneration and the expression of repair‐related genes

The neurite outgrowth experiments clearly demonstrated that the differentiated NCCs could provide trophic support and could be suitable support cell candidates in an artificial nerve graft environment. We therefore assessed the regenerative potential of the differentiated NCCs in a rat sciatic nerve injury model. Differentiated NCCs and hESCs were embedded in a fibrin matrix and seeded into the lumen of biodegradable conduits. Rat Schwann cells were also seeded in an identical manner and used as a control group to determine the regenerative efficacy of the transplanted human cells. The seeded conduits were subsequently used to bridge a 10‐mm transected sciatic nerve gap, and regeneration distance was assessed 2 weeks after transplantation.

**Figure 3:**
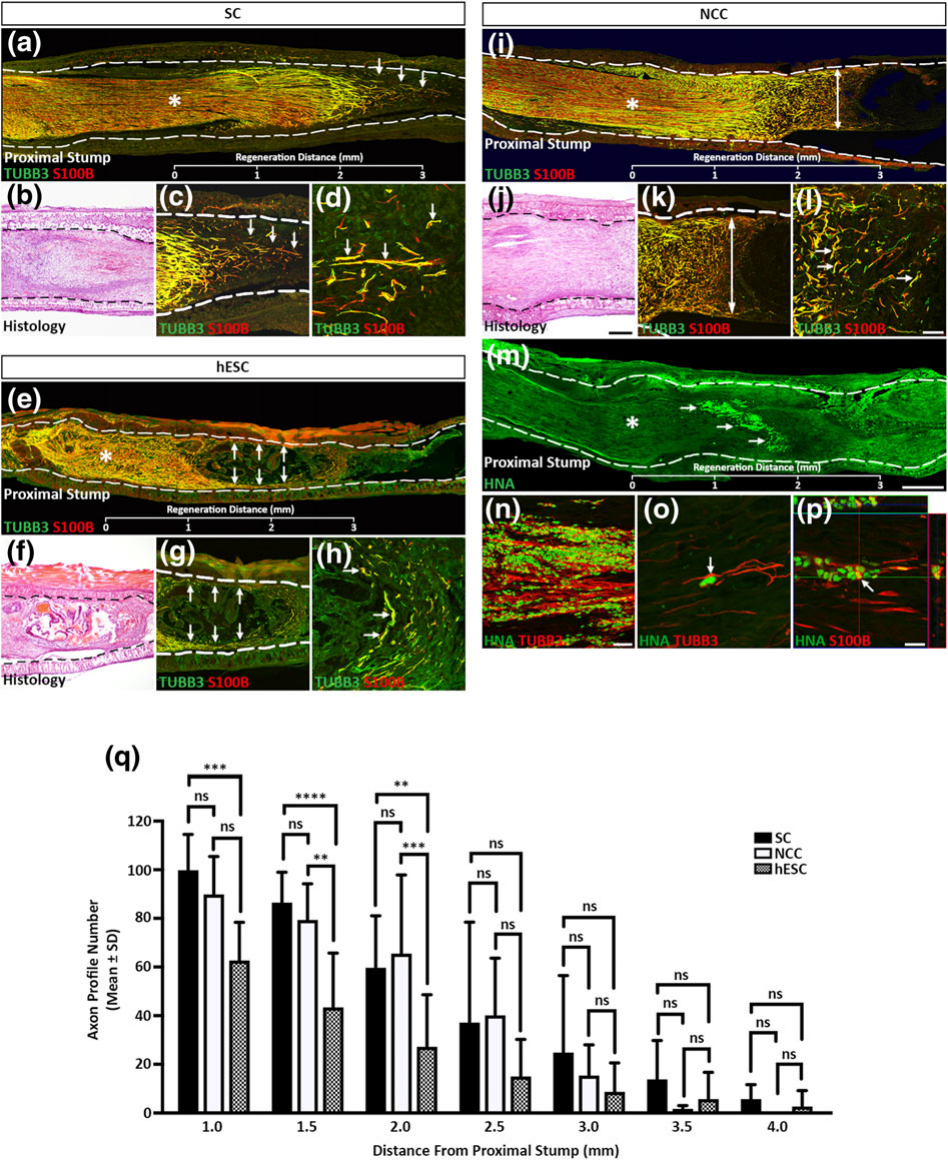
Differentiated neural crest cells (NCCs) enhance *in vivo* sciatic nerve regeneration. (a–p) Immunohistochemical and histological analyses of longitudinal sections through transplanted biodegradable conduits seeded with control rat (a–d) Schwann cells, (e–h) human embryonic stem cells (hESCs), or (i–p) differentiated NCCs. Dashed lines mark the walls of the conduits. Asterisks represent the site of nerve transection and the beginning of the regeneration front. (q) Axon profile numbers beginning 1 mm distal to the proximal stump. The data represent the mean ± *SD* of five independent measurements from each animal and condition. Scale bars: (a, e, i, and m) 500 μm, (b, f, and j) 200 μm, (c, g, k, and n) 200 μm; (d, h, and l) 50 μm, and (o–p) 20 μm. The *p* values are denoted as follows: ns = not significant; ^**^ *p* ≤ .01, ^***^ *p* ≤ .001, ^****^ *p* ≤ .0001. HNA = human nuclear antigen; SC = Schwann cells

Immunohistochemical and histological analyses of the conduits harvested from the control rat Schwann cell group demonstrated that the proximal regeneration front appeared as a single growth cone projecting centrally within the lumen of the conduit (Figure 3a–d, arrows). As expected, the conduits seeded with hESCs developed centrally positioned teratoma‐like structures with the axonal regeneration front extending along the walls of the conduit (Figure 3e–h, arrows). Contrastingly, the conduits seeded with NCCs stimulated a robust regeneration front across the entire lumen of the conduit without the elaboration of an obvious growth cone (Figure 3i–k, double arrows) with the TUBB3 positive axons lying in close proximity to S100B positive infiltrating Schwann cells (Figure 3l, arrows). Accordingly, the presence of HNA positive cells demonstrated that the differentiated NCCs were surviving in the conduit environment (Figure 3m, arrows) and were intimately associating with the TUBB3 positive regeneration front (Figure 3n and o, arrow). Furthermore, in some instances, orthogonal projection analysis demonstrated that the transplanted NCCs were both S100B and HNA positive, suggesting that some of the NCCs had further differentiated along glial lineages in the conduit environment (Figure 3p, arrow).

Axonal profile numbers were comparable between the control Schwann cells and differentiated NCC groups for a distance of up to 3 mm from the proximal stump (Figure 3q). This was in contrast to that seen for the conduits seeded with hESC where reduced numbers of proximally regenerating axons were consistently observed. Profile numbers for the differentiated NCC group were then observed to decline rapidly at distances greater than 3 mm due to the absence of a defined regeneration front. Whereas progressively reduced numbers of axons were quantified at more distal positions in the control Schwann cells conduits due to the cone‐like regeneration front observed in this treatment group.

**Figure 4:**
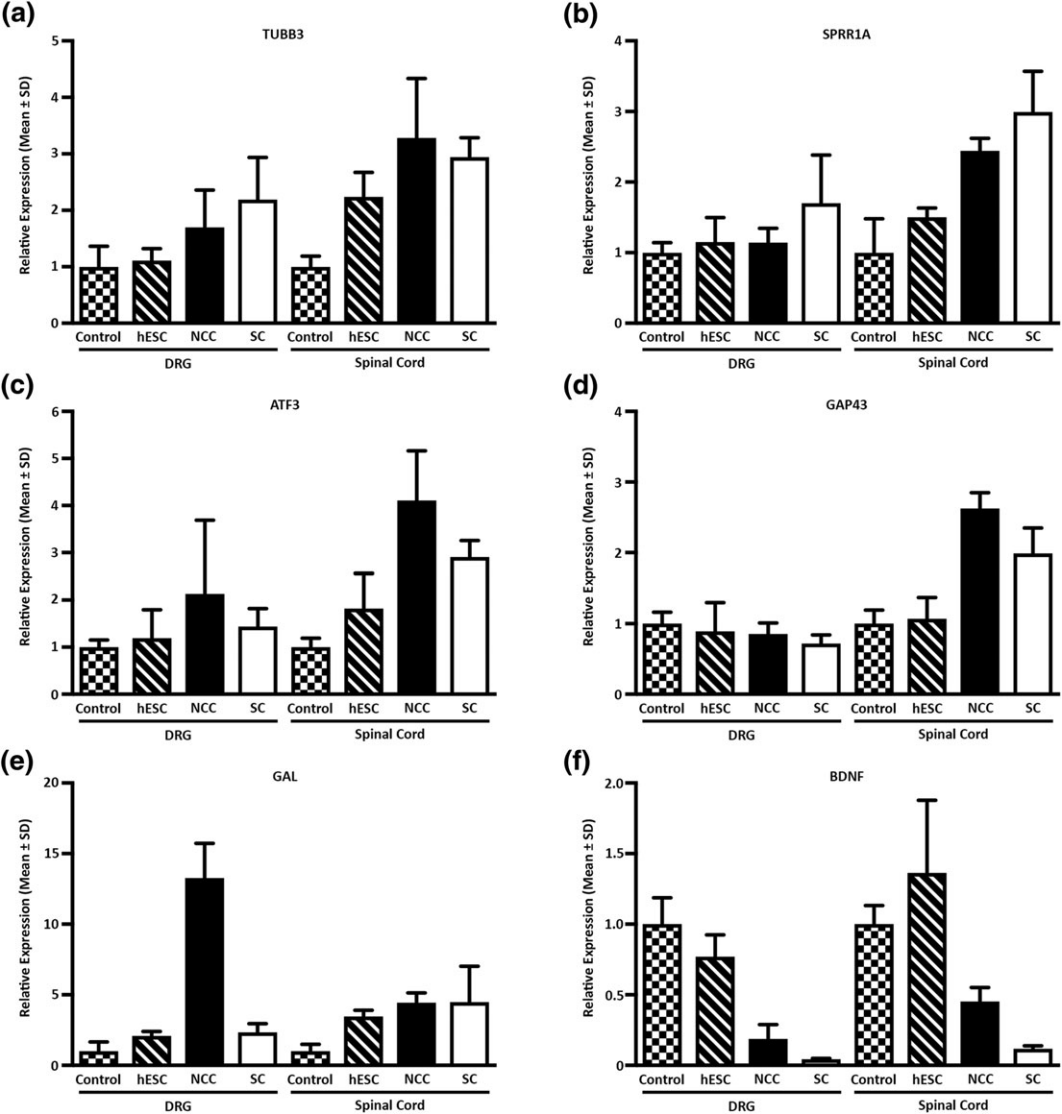
Quantitative reverse transcription–polymerase chain reaction analysis of regenerative marker gene expression in dorsal root ganglions (DRGs) and spinal cords. (a) TUBB3, (b) SPRR1A, (c) ATF3, (d) GAP43, (e) GAL, and (f) BDNF relative mRNA levels were measured in rat L4–L6 DRG and spinal cord segments. Relative expression levels are given with respect to the untreated control samples whose value has been set to 1. The data represent the mean ± *SD* of three independent measurements. hESC = human embryonic stem cells; NCC = neural crest cells; SC = Schwann cells

We next performed quantitative reverse transciption–PCR analysis on harvested ipsilateral L4–L6 DRG and corresponding spinal cord segments to assess the transcriptional activity of repair‐related genes (Figure 4). In this experiment, we focused our analysis on animals that were transplanted with conduits containing rat Schwann cells, hESCs, or differentiated NCCs and compared the results to a control group composing of injured but non‐transplanted animals. Genes that modulate axonal structure (TUBB3, 3.3‐fold increase in spinal cord) and axonal outgrowth (small proline‐rich protein 1A [SPRR1A], 2.4‐fold increase in spinal cord and activating transcription factor (ATF3), 4.1‐fold increase in spinal cord) were changed in the NCC group compared with the control group (Figure 4a–c). PNS injury triggers a highly coordinated regenerative programme involving other genes such as growth‐associated protein 43 (GAP43), Galanin (GAL), and BDNF being at the core of this response (Basi, Jacobson, Virag, Schilling, & Skene, 1987; Geremia et al., 2010; Skofitsch & Jacobowitz, 1985). We observed that these critical regenerative genes were differentially modulated within the DRG and spinal cord of animals transplanted with NCCs. Elevated levels of GAP43 (2.6‐fold, spinal cord) and GAL (13.2‐fold, DRG) were detected in the harvested tissues, whereas a parallel decrease in BDNF transcripts was observed in both DRG and spinal cord segments (Figure 4d–f).

In summary, the differentiated NCCs were able to stimulate axon regeneration in a sciatic nerve injury model. A robust regeneration front was observed across the entire lumen of the conduit with the transplanted NCCs being intimately associated with the growing axons with subsets further differentiating into glial‐like cells in the conduit environment. Moreover, the differentiated NCCs were able to stimulate the expression of several critical regeneration‐associated genes within the DRG and spinal cord of the transplanted animals.

## 4 DISCUSSION

NCCs are a migratory cell type that originate from the dorsal neural tube and represent the parent population to cell lineages within the PNS such as myelinating Schwann cells and sensory neurons (Dupin & Sommer, 2012). Despite this multipotent capability, the use of NCCs as support cells in artificial nerve graft approaches for the treatment of PNI remains unexplored. This is hampered in part due to the transient nature of NCCs, which makes their *in vivo* isolation a difficult task. The work presented in this study demonstrates that NCCs can be efficiently differentiated from hESC. The enriched NCCs exhibit enhanced secretion of several trophic factors and are able to stimulate nerve regeneration when transplanted within an experimental artificial graft.

Our work extends previous studies showing that dual‐SMAD inhibition and early WNT activation is a robust method for differentiating NCCs from hESCs (Chambers et al., 2016; Kreitzer et al., 2013). This protocol is fast and efficient and when combined with MACS results in the majority of the isolated cells expressing the established NCC markers NGFR, B3GAT1, TFAP2A, and SOX10 (Bronner‐Fraser, 1986; Kim et al., 2003; Lee et al., 2007; Nagase et al., 2003). Furthermore, the enriched NCCs exhibit multipotent capability as demonstrated by the expression of neuronal and glial markers (Blanchard et al., 2015; Kreitzer et al., 2013; Liu et al., 2012; Schrenk‐Siemens et al., 2015). Thus, the cells display all the characteristic hallmarks of bona fide NCCs (Bronner‐Fraser, 1994).

It would be interesting to compare the characteristics of NCCs generated by the dual‐SMAD approach against cells derived by other established differentiation regimes such as stromal‐derived inducing activity, embryoid body formation, and neural rosette‐derived protocols (Brokhman et al., 2008; Elkabetz et al., 2008; Jiang et al., 2009; Lee et al., 2007; Pomp et al., 2005; Pomp et al., 2008; Zhou & Snead, 2008). Cells generated by contrasting differentiation protocols may possess different self‐renewal, migratory, and lineage capabilities upon *in vivo* transplantation (Chimge & Bayarsaihan, 2010; Noisa et al., 2014). Furthermore, not all of the enriched NGFR positive cells in this study were SOX10 positive, and this may be indicative of a mixed population of both pre‐migratory and migratory NCCs. Alternatively, because single marker sorting was used, we cannot completely exclude the possibility that the enriched NGFR positive cells are predominately composed of NCCs but also smaller populations of other dorsal neural tube cells (Curchoe et al., 2010). Thus, further characterisation of our differentiation cultures is required to truly define the composition of the enriched population. This could be accomplished by single‐cell RNA sequencing because this technique would not only define cell types but also segregate the NCC population into cranial, cardiac, and trunk subtypes (Kreitzer et al., 2013).

The main function for any support cell in an artificial nerve graft environment is to secrete high levels of trophic factors that subsequently signal through receptor tyrosine kinases to coordinate the regenerative process (Abe, Borson, Gambello, Wang, & Cavalli, 2010; Christie, Webber, Martinez, Singh, & Zochodne, 2010; Fairbairn et al., 2015; Faroni, Mobasseri, Kingham, & Reid, 2015; Park, Liu, Hu, Kanter, & He, 2010; Zhou & Snider, 2006). Moreover, the use of blocking antibodies in conditioned medium experiments has established that regeneration of peripheral nerves is enhanced by the presence of active trophic factors secreted by the candidate support cell (Ribeiro‐Resende et al., 2009). We observed that medium conditioned by the differentiated NCCs exhibited elevated levels of several trophic factors including BDNF, which is known to be important for neuronal survival and axon elongation (Nakazawa, Tamai, & Mori, 2002). Regardless of the absolute trophic factor profile, our data indicate that medium conditioned by the NCCs was able to induce robust and consistent neurite outgrowth of both human SH‐SY5Y neuroblastoma and primary rat DRG neurons. The fact that NCCs naturally secreted high levels of trophic factors and support neurite outgrowth suggests that they are superior to other types of candidate support cells. For example, the nerve growth promoting behaviour of adult stem cells could only be initiated if the cells were either stimulated or even differentiated into a Schwann cell‐like lineage (Kingham et al., 2007).

The regenerative potential of the differentiated NCCs was assessed in a rat sciatic nerve injury model. The transplanted NCCs were viable and intimately associated with the regeneration front. In addition, subsets of the transplanted NCCs had differentiated into glial‐like cells in the conduit environment. These encouraging results corroborate with previous studies of NCC survival and differentiation in artificial nerve conduits (Lin et al., 2011). Moreover, no teratoma formation was seen in the NCC transplanted animals indicating the effective removal of pluripotent cells using the combination of dual‐SMAD inhibition and MACS enrichment. This observation agrees with the documented absence of teratoma formation for up to a year following NCC transplantation in a rat sciatic nerve model (Wang et al., 2011). The greatest regenerative distance was through conduits seeded with rat Schwann cells as previously described (Sinis et al., 2005). This was expected because the hosts own myelinating glia, which should create the optimal environment for axonal regeneration. However, axonal growth did overlap with Schwann cell infiltration in the NCC group, and this observation suggests that the regenerating axons were associating with myelinating glia and demonstrates that the presence of the differentiated NCCs can trigger a robust regeneration response (Chen et al., 2005). Our data therefore demonstrate that even if NCCs do not fully compare with the potential of endogenous Schwann cells, they can play an effective role by providing local trophic factor signalling at the regeneration front.

One major difference between the Schwann cell and NCC treatment groups was the appearance of the regeneration front. A regenerating cone was observed centrally within the lumen of the conduit in the Schwann cell group, whereas a robust yet disorganised regeneration front was observed across the entire lumen in the NCC transplanted animals. Previous studies have demonstrated that growing axons become trapped in areas of locally elevated levels of neurotrophic factors, and the regeneration process stalls due to the formation of nerve coils (Eggers et al., 2013; Eggers et al., 2008; Santosa et al., 2013; Tannemaat et al., 2008). Such a mechanism could explain the appearance of the regeneration front within the NCC treatment group in this current study.

A number of regeneration‐associated genes were specifically up‐regulated in the spinal cord and not in the DRG in response to the NCCs. This suggests that the NCCs might enhance motor neuron regeneration to a greater extent than sensory neurons and mirrors our previous results using adipose derived‐stem cells (Kingham et al., 2014). Our observations of elevated levels of GAP43, SPRR1A, and ATF3 are consistent with other studies showing that co‐expression of these molecules is associated with injured and regenerating neurons (Starkey et al., 2009). In contrast to these genes, the expression of GAL was more elevated in the DRG compared with the spinal cord. Given the diverse roles played by GAL in pain signalling and neurotrophic activity, the long‐term consequences of these changes will need to be delineated (Xu, Hokfelt, Bartfai, & Wiesenfeld‐Hallin, 2000).

## 5 CONCLUSION

This study describes an efficient and robust protocol for the generation of NCCs from hESCs. Dual‐SMAD inhibition coupled with MACS yields cells that resemble genetic, phenotypic, and functional characteristics of bona fide neural crest. The differentiated NCCs secrete a combination of biologically active trophic factors that stimulate peripheral nerve regeneration when incorporated into an artificial nerve graft. The promising results achieved in this study highlight the potential of differentiated NCCs as support cells for the treatment of PNI. Moreover, this pilot study acts as an entry point into longer term experiments to assess the efficacy of NCCs in promoting functional recovery. Such assessments could involve remyelination assays, nerve conduction velocity, and walking track analyses.

## CONFLICTS OF INTEREST

The authors declare no conflicts of interest.

## AUTHOR CONTRIBUTION

I. J., L. C., and P. J. K. conceived and designed the study. I. J., Li. N. N., Le. N. N., and P. J. K. collected and/or assembled the data. I. J., Le. N. N., L. C., and P. J. K. analysed and interpreted the data and wrote the manuscript. M. R. and A. U. provided the study materials. All authors gave their final approval of the manuscript. M. W. and L. C. provided salary financial support to I.J. via grants from the acknowledged study support organisations.

## Supporting information


**Figure S1.** Immunohistochemical analyses demonstrate the specificity of the human nuclear antigen antibody. Longitudinal sections through transplanted biodegradable conduits seeded with rat Schwann cells (A), hESCs (B) or differentiated NCCs (C) were stained with the HNA antibody employed in this study. HNA positive cells are only observed in conduits seeded with hESCs (B, arrows) or NCCs (C, arrows) thus demonstrating the specificity of the HNA antibody towards cells of human origin. No cross reactivity is observed in the conduit seeded with rat Schwann cells (A). Dashed lines demarcate the conduit walls. Scale bar: (A – C) 500 μm. Abbreviations: hESC, human embryonic stem cells; HNA, human nuclear antigen; NCC, neural crest cells; SC, Schwann cells.
**Table S1.** Oligonucleotide sequences and amplicon sizes used in this study.
