# Supplementary material for: Regenerative effects of human embryonic stem cell‐derived neural crest cells for treatment of peripheral nerve injury
Source: J Tissue Eng Regen Med. 2018 Feb 18;12(4):e2099–109. doi: 10.1002/term.2642 (PMC5947619; doi:10.1002/term.2642)
Supplement: Supplementary file 1 — Figure S1. Immunohistochemical analyses demonstrate the specificity of the human nuclear antigen antibody. Longitudinal sections through transplanted biodegradable conduits seeded with rat Schwann cells (A), hESCs (B) or differentiated NCCs (C) were stained with the HNA antibody employed in this study. HNA positive cells are only observed in conduits seeded with hESCs (B, arrows) or NCCs (C, arrows) thus demonstrating the specificity of the HNA antibody towards cells of human origin. No cross reactivity is observed in the conduit seeded with rat Schwann cells (A). Dashed lines demarcate the conduit walls. Scale bar: (A – C) 500 μm. Abbreviations: hESC, human embryonic stem cells; HNA, human nuclear antigen; NCC, neural crest cells; SC, Schwann cells. Table S1. Oligonucleotide sequences and amplicon sizes used in this study. [file TERM-12-e2099-s001.docx]

**Supporting Information.**

**Full Title:** Regenerative effects of human embryonic stem cell-derived neural crest cells for treatment of peripheral nerve injury.

**Short Title:** hESC-derived NCCs for peripheral nerve repair.

**Authors:** Iwan Jones^1,2^, Liudmila N. Novikova^2^, Lev N. Novikov^2^, Monika Renardy^3^, Andreas Ullrich^3^, Mikael Wiberg^2,4^, Leif Carlsson^1^ & Paul J. Kingham^2^.

**Affiliations:** ^1^Umeå Center for Molecular Medicine, Umeå University, 901 87 Umeå, Sweden; ^2^Laboratory of Neural Repair and Cellular Therapy, Department of Integrative Medical Biology, Umeå University, 901 87 Umeå, Sweden; ^3^ITV Denkendorf Productservice GmbH, Koerschtalstr. 26, D-73770 Denkendorf, Germany; ^4^Hand and Plastic Surgery, Department of Surgical and Perioperative Sciences, Umeå University, 901 85 Umeå, Sweden.

**Table 1. Oligonucleotide sequences and amplicon sizes used in this study.**

| **Gene** | **Sense Primer (5′→3′)** | **Antisense Primer (5′→3′)** | **Size (bp)** |
| --- | --- | --- | --- |
| *ANGPT1* | CTTGACCGTGAATCTGGAGC | AGCAAGACATAACAGGTGAG | 923 |
| *ATF3* | GACTGGTATTTGAAGCCAGGAGTG | GGACCGCATCTCAAAATAGC | 96 |
| *BDNF* | AGAGGCTTGACATCATTGGCTG | CAAAGGCACTTGACTACTGAGCATC | 149 |
| *GAL* | ATGCCAACAAAGGAGAAGAG | AGGTGCAAGAAACTGAGAAA | 224 |
| *GAP43* | GTCCACTTTCCTCTCTATTTC | TGTTCATTCCATCACATTGA | 131 |
| *GAPDH* | GAAGGTGAAGGTCGGAGT | CAAGCTTCCCGTTCTCAGC | 197 |
| *GDNF* | CACCAGATAAACAAATGGCAGTGC | CGACAGGTCATCATCAAAGGCG | 335 |
| *IGF1* | TGTCCTCCTCGCATCTCTTC | CACTCCCTCTACTTGCGTTC | 357 |
| *NGF* | ATACAGGCGGAACCACACTCAG | GTCCACAGTAATGTTGCGGGTC | 174 |
| *NTF3* | GGGAGATCAAAACGGGCAAC | ACAAGGCACACACACAGGAC | 222 |
| *SPRR1A* | TCCATCACCATACCAGCAGA | TAGCACAAGGCAATGGGACT | 124 |
| *TUBB3* | CCGCCTGCCTCTTCGTCTC | TAGTTGCCGCTGGGGTCTATG | 144 |
| *VEGFA* | TACCTCCACCATGCCAAGT | TGCATTCACATTTGTTGTGC | 343 |

**Abbreviations:** bp, base pair.

**
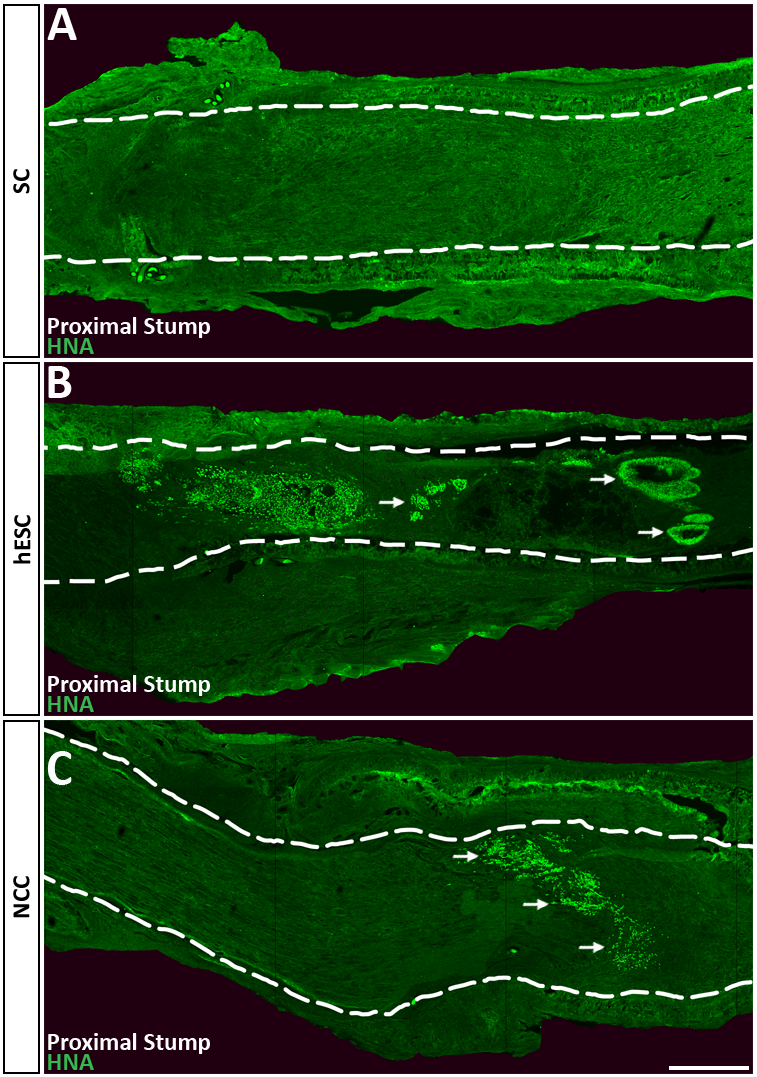
**

**Figure S1. Immunohistochemical analyses demonstrate the specificity of the human nuclear antigen antibody.** Longitudinal sections through transplanted biodegradable conduits seeded with rat Schwann cells (A), hESCs (B) or differentiated NCCs (C) were stained with the HNA antibody employed in this study. HNA positive cells are only observed in conduits seeded with hESCs (B, arrows) or NCCs (C, arrows) thus demonstrating the specificity of the HNA antibody towards cells of human origin. No cross reactivity is observed in the conduit seeded with rat Schwann cells (A). Dashed lines demarcate the conduit walls. Scale bar: (A – C) 500 μm. Abbreviations: hESC, human embryonic stem cells; HNA, human nuclear antigen; NCC, neural crest cells; SC, Schwann cells.
